# Genotyping and phylogenetic analysis of canine parvovirus circulating in Egypt

**DOI:** 10.14202/vetworld.2020.326-333

**Published:** 2020-02-19

**Authors:** Kawther Sayed Zaher, Wahid Hussein El-Dabae, Mostafa Mohamed El-Sebelgy, Naglaa Ibrahim Aly, Zeinab Taha Salama

**Affiliations:** 1 Department of Microbiology and Immunology, Veterinary Research Division, National Research Centre, Dokki 12622, Giza, Egypt; 2 Department of Pet Animal Vaccine Research, Veterinary Serum and Vaccine Research Institute, Abbasia, Egypt

**Keywords:** canine parvovirus, Egypt, genotyping, phylogenetic analysis, serotyping

## Abstract

**Aim:**

This study aimed to detect and characterize current genotypes of canine parvovirus (CPV) in Egypt during 2018.

**Materials and Methods:**

A total of 50 fecal swabs were collected from clinically infected domestic dogs of 2-5 months of age, suspected to suffer from CPV infection, from Cairo and Giza Governorates. The samples were subjected to qualitative antigen detection using the rapid test, followed by isolation on Madin-Darby Canine Kidney (MDCK) cells, molecular characterization with partial amplification of VP2 gene using polymerase chain reaction (PCR), followed by sequencing and phylogenetic analysis.

**Results:**

Out of 50 fecal samples, 20 samples were positive (40%) by Rapid CPV/canine coronavirus Ag Test Kit. These positive samples were cultured successfully on MDCK cells. Nine randomly chosen samples out of 30 apparently negative samples were amplified using PCR with primers Hfor and Hrev to yield a typical 630 bp fragment. Then, six randomly chosen samples out of nine were amplified using PCR with primers Pbs and Pbas to yield a typical 427 bp fragment. Sequencing, BLAST analysis and assembly of the two fragments (630 bp and 427 bp) to produce 912 bp fragments, in the six samples, revealed two serotypes CPV-2b and CPV-2c. The obtained strains were submitted to GenBank and given accession numbers MK642272, MK642273, MK642274, MK642275, MK642276, and MK642277. Phylogenetic analysis of the Egyptian strains serotype 2b illustrated that they were closely related to Thailand strains (accession numbers KP715709, KP715694, KP715701, and KP715700); while Egyptian strains serotype 2c was closely related to Thailand strains (accession numbers MH711894 and MH711902), Taiwanese strain (KU244254), Chinese strain (MF467242), and Vietnamese strain (accession number LC216910).

**Conclusion:**

The current research recommends further epidemiological studies to assess the extent of the occurrence of different serotypes of CPV in Egypt and the efficiency of imported and locally produced vaccines in protection against CPV infection.

## Introduction

Canine parvovirus type 2 (CPV2) is one of the most important global pandemic contagious viral diseases affecting canine domestic population, especially young puppies causing acute hemorrhagic enteritis, myocarditis, vomition, and immunosuppression [1]. CPV2 has a small diameter (about 25 nm), enveloped with an icosahedral capsid. CPV2 is classified within the family *Parvoviridae*, subfamily *Parvovirinae*, genus *Protoparvovirus*, and species *carnivore protoparvovirus 1* [2]. The viral genome is a single-stranded, linear, negative-sense DNA comprising about 5200 nucleotides. The genome encodes four proteins; two non-structural proteins called NS1 (involved in viral replication) and NS2 (has a role in capsid assembly) and two structural proteins termed VP1 (involved in cell infection) and VP2 (forms the viral capsid and is the main protective antigen) [3].

The first recognition of this virus was in the 70s, as a novel virus among the domestic canines. The virus was designated as CPV2, at that time, due to the existence of minute virus of canine, known as CPV1 [4]. During the 80s, the original virus (CPV2) circulating globally acquired mutations, which led to the emergence of two antigenic subtypes (CPV2a and CPV2b) and replacement of the prototype CPV2 with the appearance of an additional antigenic subtype (CPV2c) in 2000 in Italy [5].

In Egypt, the virus was initially reported in 1982, in military police dogs showing clinical manifestations, pathological outcomes [6] and CPV antibodies were detected by the serological study in spring months [7]. In 2005, a Penta Dog inactivated cell culture vaccine containing CPV was prepared [8]. In addition, the effect of different adjuvants on the inactivated canine parvo vaccine was investigated [9]. Egg yolk (IgY) was prepared against CPV conjugated with fluorescein isothiocyanate and horseradish peroxidase. The CPV was adapted among other canine viruses on Madin-Darby Canine Kidney (MDCK) cells without serum and oral vaccination of puppies was formulated with canine core vaccine including parvovirus [10,11]. Isolation of CPV2 on Vero cells, polymerase chain reaction (PCR), and sequence analysis confirmed the presence of genotype CPV2b in 2012. While in 2014, clustering of the virus within genotypes 2b and 2c was observed [12,13]. Antibodies detected against CPV in domestic dogs, using serodiagnosis, demonstrated high susceptibility of 4-month-old puppies in 2016. In 2018, genotypes 2a and 2b were identified using genetic characterization with special reference to multiple mutations in genotype 2b [14,15]. Genotype 2b is still circulating in Egypt with successful isolation of virus on Vero cells [16].

Research articles regarding CPV are few in number in Egypt and so an in-depth insight is needed to highlight the genotypes circulating in Egypt and this research is an attempt to contribute to this goal. There are many reports concerning the infection of vaccinated dogs, which urges further investigation about the efficacy of the available vaccines. This study aimed to detect and characterize current genotypes of CPV in Egypt during 2018.

## Materials and Methods

### Ethical approval

This study was approved by Ethical Committee for Medical Research at the National Research Centre, Egypt and in accordance with local laws and regulations.

### Samples

A total of 50 fecal swabs were collected from Cairo and Giza Governorates during 2018 from clinically infected domestic dogs of 2-5 months of age (native breed and German Shepherd) showing moderate-to-severe gastrointestinal signs including bloody diarrhea, dehydration, vomition, inappetence, and lethargy with no history of previous vaccination.

Fecal swabs were collected (separately from each animal) in labeled tubes containing phosphate-buffered saline (PBS) with 10% of antibiotic solution and subjected to two cycles of freezing and thawing then for centrifugation at 2000 rpm for 10 min. The supernatant fluid was separated and kept at −80ºC until used for virus isolation [16].

### Qualitative detection of CPV antigens in feces of dogs

All of the collected fecal swabs were tested for CPV Ag using Rapid CPV/canine coronavirus [CCV] Ag Test Kit (Cat. No. RC1105DD), Bionote, Republic of Korea (according to manufacturer’s instructions).

### Isolation of CPV on MDCK

Fecal swabs obtained from clinically infected domestic dogs were washed with 1 ml PBS and centrifuged at 10,000× *g* for 5 min/4°C. The positive samples for CPV antigens, using Rapid CPV/CCV Ag Test Kit, were filtered using a 0.22 μm syringe filter. The MDCK cell line was grown to confluence in minimal essential medium (MEM) (Sigma-Aldrich) containing 10% fetal calf serum (Sigma-Aldrich) at 37°C with 5% CO_2_. When the monolayers were 80-90% confluent, the growth medium was decanted and 0.1 ml of viral inoculum was added to a 25 cm^2^ tissue culture flask. Simultaneous inoculations of similar flasks with an equal volume of sterile PBS served as negative culture controls. Maintenance of cell cultures was done using MEM with 1% fetal calf serum. The inoculum was allowed to adsorb at 37°C for 1 h. After 1 h, the inoculum was pipetted out and the monolayer was washed with PBS. Finally, MEM was added to each monolayer including the controls then incubated at 37°C. The monolayer was examined daily for the appearance of cytopathic effects (CPEs) [17,18].

### PCR for isolated virus

#### DNA extraction

DNA extraction from samples was performed using the QIAamp DNA Mini Kit Cat. No. 51304 (Qiagen GmbH, Germany) according to manufacturer’s instructions. Briefly, 200 µl of the samples suspension were incubated with 10 µl of proteinase K and 200 µl of lysis buffer at 56°C for 10 min. After incubation, 200 µl of absolute ethanol was added to the lysate. The samples were washed and centrifuged with reference to the manufacturer’s recommendations. Nucleic acid was eluted with 100 µl of elution buffer provided with the kit.

#### Oligonucleotide primers

Primers listed in Table-1 were supplied from Metabion (Germany).

**Table-1:** Primers sequences, target genes, amplicon sizes, and polymerase chain reaction cycling conditions.

Target gene	Primers sequences	Amplified segment (bp)	Initial denaturation	Amplification (35 cycles)			Final extension	Reference

				Denaturation	Annealing	Extension		
*Vp2*	Hfor: CAGGTGA TGAATTTGCTACA	630	94°C 5 min	94°C 30 s	55°C 40 s	72°C 45 s	72°C 10 min	[20]
	Hrev: CATTTGGA TAAACTGGTGGT							
	Pbs: CTTTAACC TTCCTGTAACAG Pbas: CATAGTTA AATTGGTTATCTAC	427	94°C 5 min	94°C 30 s	55°C 40 s	72°C 40 s	72°C 10 min	

#### PCR amplification

Primers were utilized in a 25 µl reaction containing 12.5 µl of EmeraldAmp Max PCR Master Mix Cat. No. RR320A (Takara, Japan), 1 µl of each primer of 20 pmol concentration, 7.5 µl of water, and 3 µl of DNA template. The reaction was performed in an Applied Biosystems 2720 thermal cycler.

### Analysis of the PCR products

PCR products were separated by electrophoresis on 1.5% agarose gel (AppliChem GmbH, Germany) in 1× TBE buffer at room temperature using gradients of 5 V/cm. For gel analysis, 15 µl of the product was loaded in each gel slot. A GeneRuler™ 100 bp ladder (Fermentas, Germany) and GelPilot^®^ 100 bp ladder (Qiagen GmbH, Germany) were used to determine the fragment sizes. The gel was photographed by a gel documentation system (Alpha Innotech, Biometra, Germany) and the data were analyzed through computer software.

### Sequence analysis of the PCR products

PCR products were purified using QIAquick PCR Product extraction kit, Cat. No. 28104 (Qiagen, Valencia) according to the manufacturer’s instructions. BigDye™ Terminator v3.1 cycle sequencing kit, Cat. No. 4337455 (PerkinElmer) was used for the sequence reaction and then purified using Invitrogen™ Centri-Sep™ Spin Columns. DNA sequences were obtained using Applied Biosystems 3130 genetic analyzer (HITACHI, Japan) in Elim Biopharmaceuticals Inc., CA, USA. A BLAST^®^ analysis (Basic Local Alignment Search Tool) was initially used to establish sequence identity to GenBank accession numbers [19]. The evolutionary history was inferred using the neighbor-joining method [20]. The optimal tree with the sum of branch length = 0.03463035 is shown. The tree is drawn to scale, with branch lengths in the same units as those of the evolutionary distances used to infer the phylogenetic tree. The evolutionary distances were computed using the p-distance method [21] and are in the units of the number of base differences per site. The rate variation among sites was modeled with a gamma distribution (shape parameter = 1). This analysis involved 30 nucleotide sequences. Codon positions included were 1^st^ + 2^nd^ + 3^rd^ + non-coding. All ambiguous positions were removed for each sequence pair (pairwise deletion option). There were a total of 912 positions in the final dataset. Evolutionary analyses were conducted in MEGA X [22].

## Results and Discussion

In this study, suspected cases of non-vaccinated CPV including native breed (mixed breed) and German Shepherd (pure breed) of age 2-5 months were investigated. Out of 50 fecal swabs tested using Rapid CPV/CCV Ag Test Kit, 20 samples were positive (40% of total samples). This test is the most commonly used field diagnostic tool because it is rapid, simple and can be carried out by both veterinarians and owners. However, the sensitivity of this test does not exceed 50% due to large amount of viral antigen required to produce strong visible band. This confirms the high specificity and low sensitivity of the test as previously declared [16].

**Figure-1:**
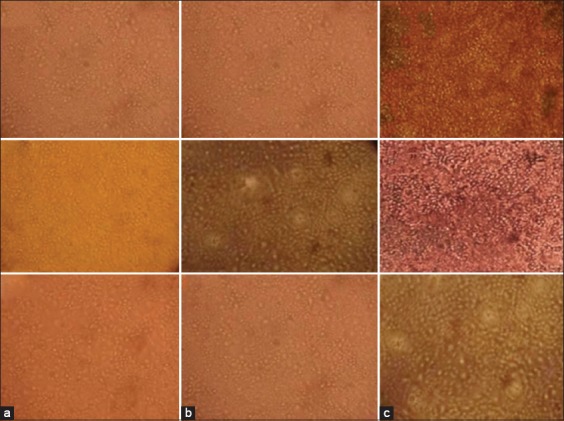
Cytopathic effects produced on Madin-Darby Canine Kidney cells by canine parvovirus, (a) negative control cell monolayer, (b) cell monolayer 2-3 days post-inoculation showing rounding and detachment of cells, and (c) cell monolayer 4-6 days post-inoculation showing rounding and detachment of cells.

The rapid kit positive samples were successfully cultured on MDCK cells for three successive passages showing typical CPE of rounding and detachment of cells on 2-3 and 4-6 days’ time mark along with negative control, Figure-1. Furthermore, CPV was adapted on MDCK cells with characteristic CPE only after the 5^th^ passage, whereas CPE at the 7^th^ passage was characterized by rounding and shortening of cells [18].

**Figure-2:**
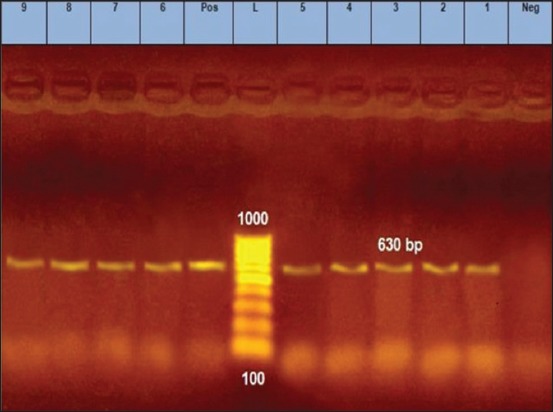
Agarose gel 1.5% (AppliChem) showing the polymerase chain reaction amplification products of expected fragment of 630 bp (lanes 1-9 samples). Lane L represents 100 bp DNA ladder (GeneRuler™, Fermentas). Lane Neg represents the negative control. Lane Pos represents the positive control.

**Figure-3:**
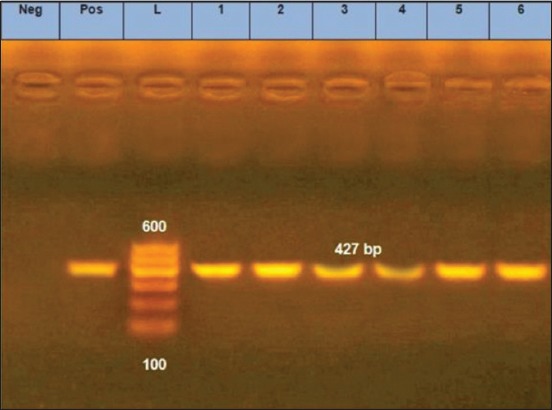
Agarose gel 1.5% (AppliChem) showing the polymerase chain reaction amplification products of expected fragment of 427 bp (lanes 1-6 samples). Lane L represents 100 bp DNA ladder (GelPilot^®^, Qiagen). Lane Neg represents the negative control. Lane Pos represents the positive control.

The nine randomly selected samples from the apparently negative ones were amplified using conventional PCR with primers Hfor and Hrev, Table-1. They were all shown to be positive at 630 bp mark, Figure-2. The first fragment was used to assure the presence of CPV in those samples. Then, six randomly chosen samples out of the nine were amplified using conventional PCR with primers Pbs and Pbas, Table-1 [20]. All tested samples were positive with a typical fragment at 427 bp, Figure-3. The positivity of the randomly chosen samples confirms that molecular-based tool (PCR) is much more sensitive than immunochromatographic method (rapid test) [16]. Similarly, traditional and nested PCRs were used to detect CPV with 81.63% (40/49) and 97.96% (48/49), respectively [17].

**Figure-4:**
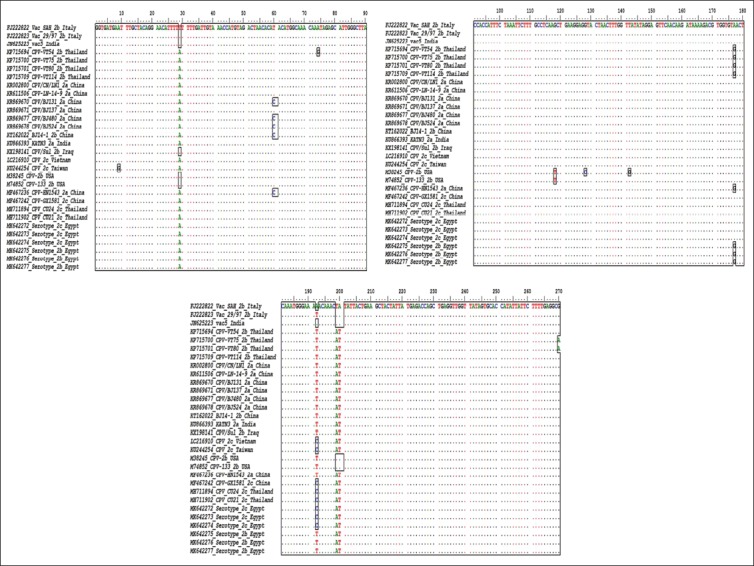
Multiple nucleotide sequence alignment of the obtained canine parvovirus Egyptian strains in comparison with different known global strains, generated by BioEdit program version 7.0.5.3. The similarities are shown as dots while differences are shown as letters and/or boxes. [Additional figures can be available from the corresponding author].

**Figure-5:**
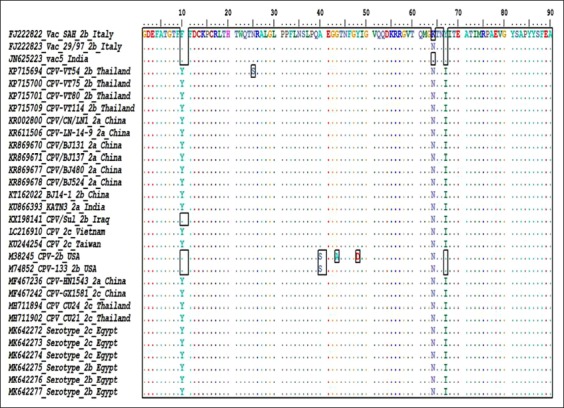
Multiple amino acid sequence alignment of the obtained canine parvovirus Egyptian strains in comparison with different known global strains, generated by BioEdit program version 7.0.5.3. Similarities are shown as dots while differences are shown as letters and/or boxes. [Additional figures can be available from the corresponding author].

Those six positive samples’ fragments were sequenced and assembled along with same positive samples of the 630 bp fragment to produce a 912 bp fragment. The sequence undergone BLAST analysis for further confirmation and for establishing similarity with other strains (Table-2). Multiple sequence alignment for both nucleotides and amino acids revealed multiple mutations, as shown in Figures-4 and 5.

**Table-2:** Some strains used in phylogenetic tree including the designation of the obtained Egyptian strains.

No.	Accession number	Strain designation	Serotype	Country of origin
1.	MK642272	NRC/Egy1/2019	2c	Egypt
2.	MK642273	NRC/Egy2/2019	2c	Egypt
3.	MK642274	NRC/Egy3/2019	2c	Egypt
4.	MK642275	NRC/Egy4/2019	2b	Egypt
5.	MK642276	NRC/Egy5/2019	2b	Egypt
6.	MK642277	NRC/Egy6/2019	2b	Egypt
7.	MH711902	CPV 2c strain CU21	2c	Thailand
8.	MH711894	CPV 2c strain CU24	2c	Thailand
9.	KR869671	CPV 2a strain CPV/BJ137 VP2 protein gene	2a	China
10.	KP715701	CPV isolate CPV-VT80 VP2 protein gene	2b	Thailand
11.	KP715700	CPV isolate CPV-VT75 VP2 protein gene	2b	Thailand
12.	KP715694	CPV isolate CPV-VT54 VP2 protein gene	2b	Thailand
13.	KP715709	CPV isolate CPV-VT114 VP2 protein gene	2b	Thailand
14.	KU244254	CPV 2c capsid protein (VP2) gene, complete cds	2c	Taiwan
15.	LC216910	CPV 2c VP2 gene for viral protein 2, complete cds, strain: CPV/dog/HCM/20/2013	2c	Vietnam
16.	MF467242	CPV isolate CPV-GX1581 VP2 protein gene, complete cds	2c	China

CPV=Canine parvovirus

The obtained six sequences were submitted to GenBank and given the following accession numbers: MK642272 (2c), MK642273 (2c), MK642274 (2c), MK642275 (2b), MK642276 (2b), and MK642277 (2b). VP2 426Asp is another name for CPV-2b and VP2 426Glu is another name for CPV-2c and this nomenclature is according to mutations/substitutions in VP2 capsid protein at residue 426. The obtained multiple amino acid alignments, Figure-5, at residue 169, which is equivalent to residue 426, for serotype 2b, aspartic acid (D) designates CPV-2b, and for serotype 2c, glutamic acid (E) designates CPV-2c [4] and this was the basis of serotyping.

**Figure-6:**
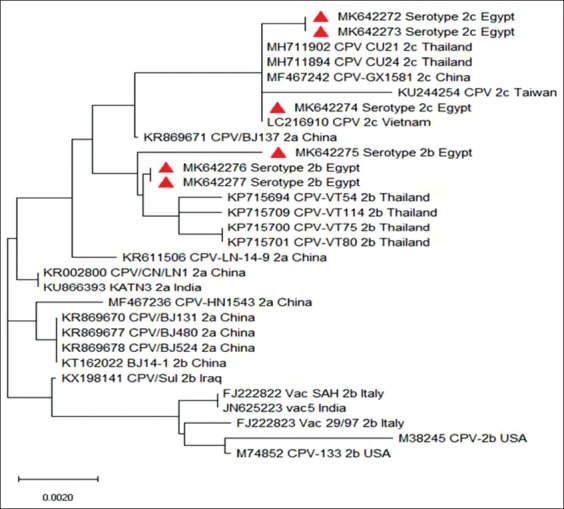
Phylogenetic analysis created by MEGA X version 10.0.5 showing clustering of Egyptian canine parvovirus strains with Thailand, Chinese, Taiwanese, and Vietnamese strains.

The phylogenetic analysis revealed that the Egyptian strains serotype 2b was clustered with the Thailand strains (KP715701, KP715700, KP715700, and KP715694), as shown in Figure-6. The same finding was clear in 2018 in an Egyptian 2b strain [16]. The Egyptian strains serotype 2c was clustered with the Thailand strains (accession numbers MH711894 and MH711902), Taiwanese strain (KU244254), Chinese strain (MF467242), and Vietnamese strain (LC216910).

**Figure-7:**
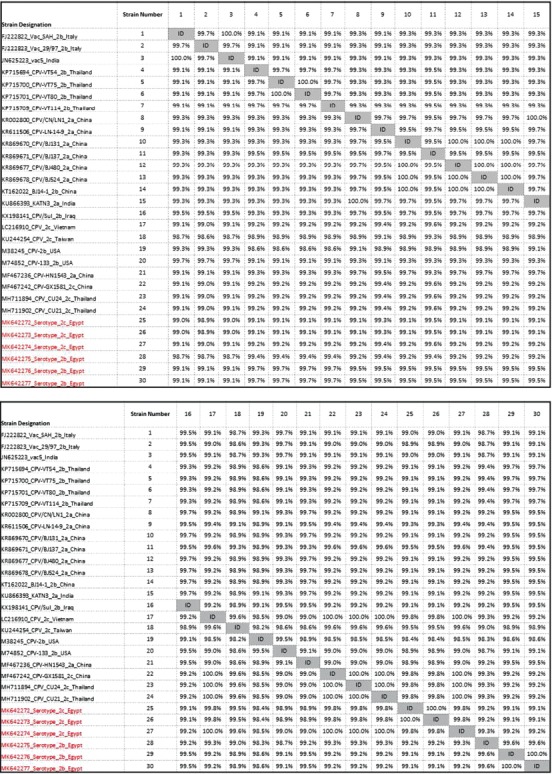
Sequence identity matrix generated by BioEdit program version 7.0.5.3. showing identity percentage between the Egyptian canine parvovirus strains and other global known strains.

The strong interrelationship with Thailand, Chinese, Vietnamese, and Taiwanese strains was further emphasized from the sequence identity matrix (Figure-7). The Egyptian CPV 2c strain (MK642274) was identical (100%) to Chinese strain (MF467242), Thailand strains (accession numbers MH711894 and MH711902), and Taiwanese strain (KU244254), while identity percentage to Vietnamese strain (LC216910) was 99%. The Egyptian CPV 2c strains (MK642272 and MK642273) were similar (99.8%) to Chinese strain (MF467242) and Thailand strains (accession numbers MH711894 and MH711902); Vietnamese strain (LC216910) was 98.9%, while the identity percentage to Taiwanese strain (KU244254) was 99.5%. The Egyptian CPV 2b strain (MK642275) was similar (99.4%) to Thailand strains (accession numbers KP715694, KP715700, KP715701, and KP715709), while the Egyptian CPV 2b strains (MK642276 and MK642277) were similar (99.7%) to the same Thailand strains.

This outcome raises the question about the epidemiological origin of the Egyptian strains and their strong relation to some Southeastern Asian countries (Thailand and Vietnam), China, and Taiwan.

## Conclusion

The parvovirus infection is strongly prevalent in Egypt with all its known serotypes; 2a, 2b, and 2c. This study was carried out to identify the prevalence of each serotype, determining the dominant one and mutations. Most Egyptian studies have a limited number of samples, which are not enough to establish realistic epidemiological data. The serotypes and mutations identified throughout this study might provide evidence for the inadequate protection of some commonly used and produced vaccines in Egypt.

The cross-protection between different serotypes in vaccination and its extent is still a debatable issue worldwide and very difficult to evaluate; as some claim that CPV-2 can protect against new antigenic types including latest type (CPV-2c), some claim that CPV-2b, during challenging, can protect against virulent field strain CPV-2c, and others that current CPV vaccines failed to protect against field strains. Vaccines should include the prevailing antigenic types of a field virus to provide complete protection. Caution should be given during vaccine development to avoid mismatch between the vaccinal strain (modified live vaccine) and infecting strain that can lead to increase the risk of an outbreak. This research reports mutation in parvovirus (DNA virus) that is not commonly found in this type of virus.

## Authors’ Contributions

NIA and ZTS collected the samples. KSZ and NIA planned this work; ZTS, WHE, and MME carried out the practical work. All authors read and approved the final manuscript.
